# Expansion of anti-AFP Th1 and Tc1 responses in hepatocellular carcinoma occur in different stages of disease

**DOI:** 10.1038/sj.bjc.6605526

**Published:** 2010-01-19

**Authors:** S Behboudi, A Alisa, S Boswell, J Anastassiou, A A Pathan, R Williams

**Affiliations:** 1 Institute of Hepatology, University College London, 69–75 Chenies Mews, London, WC1E 6HX, UK; 2 Laboratory of Human Immunology and Infection, School of Health Sciences and Social Care, Brunel University, London, Uxbridge UB8 3PH, UK

**Keywords:** AFP, HCC, stage of disease, Okuda, Child–Pugh, IFN-*γ*-producing cells

## Abstract

**Background:**

*α*-Fetoprotein (AFP) is a tumour-associated antigen in hepatocellular carcinoma (HCC) and is a target for immunotherapy. However, there is little information on the pattern of CD4 (Th1) and CD8 (Tc1) T-cell response to AFP in patients with HCC and their association with the clinical characteristics of patients.

**Methods:**

We therefore analysed CD4 and CD8 T-cell responses to a panel of AFP-derived peptides in a total of 31 HCC patients and 14 controls, using an intracellular cytokine assay for IFN-*γ*.

**Results:**

Anti-AFP Tc1 responses were detected in 28.5% of controls, as well as in 25% of HCC patients with Okuda I (early tumour stage) and in 31.6% of HCC patients with stage II or III (late tumour stages). An anti-AFP Th1 response was detected only in HCC patients (58.3% with Okuda stage I tumours and 15.8% with Okuda stage II or III tumours). Anti-AFP Th1 response was mainly detected in HCC patients who had normal or mildly elevated serum AFP concentrations (*P*=0.00188), whereas there was no significant difference between serum AFP concentrations in these patients and the presence of an anti-AFP Tc1 response. A Th1 response was detected in 44% of HCC patients with a Child–Pugh A score (early stage of cirrhosis), whereas this was detected in only 15% with a B or C score (late-stage cirrhosis). In contrast, a Tc1 response was detected in 17% of HCC patients with a Child–Pugh A score and in 46% with a B or C score.

**Conclusion:**

These results suggest that anti-AFP Th1 responses are more likely to be present in patients who are in an early stage of disease (for both tumour stage and liver cirrhosis), whereas anti-AFP Tc1 responses are more likely to be present in patients with late-stage liver cirrhosis. Therefore, these data provide valuable information for the design of vaccination strategies against HCC.

Hepatocellular carcinoma (HCC) overexpresses several tumour-associated antigens (Kittaka *et al*, 2008). Some of these antigens, such as MAGE, Glypican-3 and NY-ESO-1, are also expressed by many other types of cancer cells (Shang *et al*, 2004; Capurro and Filmus, 2005), whereas *α*-fetoprotein (AFP) is specific to HCC and testicular carcinoma. The synthesis of AFP decreases dramatically after birth and only trace amounts are expressed in the adult liver. During liver regeneration and hepatocarcinogenesis, expression of the *AFP* gene is reactivated in adults (Cooper *et al*, 2001; Mizejewski, 2007), with the majority of HCC patients showing an increase in serum AFP levels. The induction of an anti-AFP cell-mediated immune response can control tumour growth in animal models (Grimm *et al*, 2000). In humans, it has been shown that B and T cells can recognise peptide epitopes within the AFP sequence and can develop into effector and/or regulatory lymphocytes. Several AFP-derived HLA class I (HLA-A2 and HLA-A24) and class II (HLA-DR) restricted CD4 (Th1) and CD8 (Tc1) epitopes have been identified (Bei *et al*, 1999; Butterfield *et al*, 1999, 2001; Meng *et al*, 2000; Alisa *et al*, 2005, 2008; Mizukoshi *et al*, 2005; Liu *et al*, 2006; Ayaru *et al*, 2007). The results from several studies show that the detection of AFP-specific CD8 T cells is restricted to HCC patients, but does not correlate with elevated serum AFP concentrations, vascular invasion or type of viral infection (Ritter *et al*, 2004; Butterfield *et al*, 2007; Thimme *et al*, 2008). In contrast, we have shown that CD4 T-cell responses to an immunodominant AFP-derived peptide are only detectable in HCC patients, and this response is mainly detected in patients with low serum AFP concentrations and early-stage disease (Alisa *et al*, 2005). To address these conflicting results, we analysed Th1 and Tc1 responses in parallel in the same group of patients/controls. Our findings demonstrate that a Th1 response is only detectable in HCC patients, whereas a Tc1 response is detectable in both HCC and controls. Moreover, these results suggest that anti-AFP Th1 responses are more likely to be present in an early tumour stage or liver cirrhosis, whereas anti-AFP Tc1 responses are more likely to be present in patients with more severe liver cirrhosis.

## Materials and methods

### Synthetic peptides

Peptides corresponding to the sequence of AFP were purchased from Mimotopes Pty Ltd. (Clayton Victoria, Australia).

### Patients

This study was approved by ethical committees and all patients gave written informed consent. Peripheral blood mononuclear cells (PBMCs) were isolated from the blood of patients with HCC or from healthy donors.

### Expansion of T cells *in vitro*

The RPMI 1640 medium, MEM medium, penicillin and streptomycin and 10% heat-inactivated FCS were purchased from Life Technologies (Grand Island, NY, USA). T-cell lines were generated as described previously (Alisa *et al*, 2005). In brief, PBMCs were re-suspended at a concentration of 1.5 × 10^6^ per ml in MEM, 10% FCS (Life Technologies), and stimulated with individual peptides (1 *μ*M) or peptide pools in 96-well plates. Recombinant IL-2 (50 IU ml^−1^) was added on day 3 of culture, and cells were analysed after a total of 10–12 days of culture.

### Intracellular IFN-*γ* staining

Expanded T cells were incubated for 5 h at 37°C at 1 × 106 cells per ml in MEM, 10% FCS with AFP-derived peptides (1 *μ*M) and in the presence of Brefeldin A (10 *μ*g ml^−1^ Sigma-Aldrich, St Louis, MO, USA). Cells were surface stained with Cy-chrome-conjugated anti-CD4 or anti-CD8 antibodies (BD PharMingen, Cowley, UK). The cells were then fixed and permeabilised using Cytofix/Cytoperm (BD PharMingen), and stained for intracellular cytokines with FITC-conjugated anti-IFN*γ* or isotype control (R&D Systems, Abingdon, UK), washed twice and analysed by flow cytometry. The expansion of T cells did not induce non-specific (no peptide) IFN-*γ*-producing T cells and all background levels (no peptide or irrelevant peptide) were between 0.1 and 0.5%. An immunological response/responder was defined as a five-fold increase in the frequency of cytokine-producing cells above control peptides/no peptide.

### AFP measurement

Concentrations of serum AFP were measured using a microparticle enzyme immunoassay kit obtained from Abbott Laboratories (Abbott Park, IL, USA) and carried out according to the manufacturer's instructions. In brief, anti-AFP microparticles were incubated with the blood specimen, and an aliquot of the reaction mixture was transferred to the matrix cell. The matrix cell was washed, and the anti-AFP conjugate was added to it. The substrate was then added to the matrix cell and the fluorescent product was measured by the microparticle enzyme immunoassay optical assembly.

### Statistical analysis

The Mann–Whitney *U-*test was used to compare the concentration of serum AFP in HCC patients and the ability of T cells to respond to the identified epitope (responder *vs* non-responder). The *χ*^2^ test was used to compare whether the percentage of CD4 T-cell responders was predominantly in patients with Okuda tumour stage I or II+III. Statistical significance was defined as *P*⩽0.05.

## Results

### An anti-AFP Tc1 response was detected in controls and HCC patients, whereas an anti-AFP Th1 response was detected only in HCC patients

**Figure 1:**
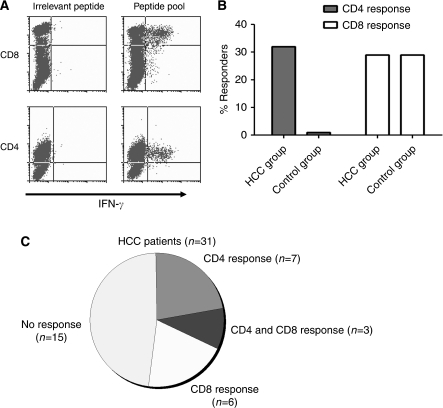
Anti-AFP CD4 T-cell responses are only detected in HCC patients. (**A**) Peptide-specific intracellular IFN-*γ* production by CD4 and CD8 T cells was determined using flow cytometry. Representatives of CD4 and CD8 T-cell responses against AFP-derived peptides are shown. (**B**) The percentages of HCC patients or controls with CD4 or/and CD8 T cells reacting to AFP-derived peptides are shown. (**C**) The number of HCC patients with or without anti-AFP CD4, CD8 and CD4 and CD8 T-cell responses is presented.

Peripheral blood mononuclear cells were isolated from a total of 31 patients with HCC (HCC01-HCC31), from 8 patients with cirrhosis (LC01-08) and from 6 healthy individuals (NC01-06) (Tables 1 and 2). T cells were expanded in culture in the presence of AFP-derived peptide pools (Supplementary Table 1). The reactivity of T-cell lines to relevant and irrelevant peptide pools was analysed using an intracellular cytokine assay for IFN-*γ* after 10–12 days of culture (Figure 1A). An anti-AFP CD4 T-cell response was detected in 32% of HCC patients (10 out of 31), whereas no CD4 T-cell response was detected in controls. In contrast, an anti-AFP CD8 T-cell response was detected in 29% of HCC patients (9 out of 31) and in 29% of the control group (4 out of 14, Figure 1B). The presence of both a CD4 and a CD8 T-cell response to AFP peptides was detected in three HCC patients (Figure 1C).

**Figure 2:**
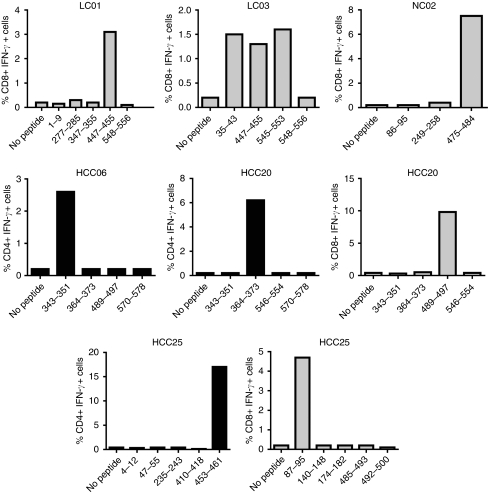
Multi-specific CD4 and CD8 T-cell responses in HCC patients and controls. Expanded T cells were incubated with indicated AFP peptides and peptide-specific intracellular IFN*γ* was analysed using flow cytometry. Naturally occurring CD4 and CD8 T-cell responses against individual AFP-derived peptides were analysed in three patients with HCC and in three controls. The percentages of peptide-specific CD4 (black) or CD8 (grey) T-cell responses to individual peptides are shown.

To determine the reacting peptide(s) within the peptide pools, expanded T cells were treated with individual peptides of the reacting pools, and peptide-specific intracellular IFN-*γ* production by CD4 and CD8 T cells was analysed using flow cytometry (Figure 2). Among the responders, PBMCs were available from LC01, LC03, NC02, HCC06, HCC20 and HCC25 for further studies. Dot plots of anti-AFP-specific IFN-*γ-*producing CD4 or CD8 T cells from these individuals are shown (Supplementary Figures 1 and 2). These results suggest that Th1 and Tc1 responses are multi-specific.

### Anti-AFP Th1 responses are mainly detected in an early tumour stage

**Figure 3:**
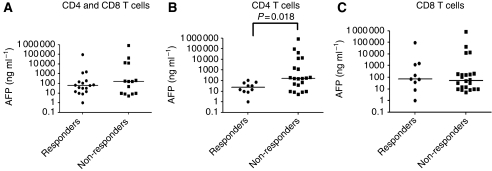
HCC patients with anti-AFP CD4 T-cells responses have significantly lower levels of serum AFP. Serum AFP levels in HCC patients with or without (**A**) anti-AFP total T-cells responses (both CD4 and CD8), (**B**) CD4 T-cell responses or (**C**) CD8 T-cell responses are shown. Anti-AFP CD4 T-cell response was detected in patients with low or moderately elevated serum AFP (*n*=0.018). Median of serum AFP levels for responders and non-responders are presented and each dot represents a patient with HCC.

An AFP-specific CD4 T-cell response was mainly detected in HCC patients with normal or mildly elevated serum AFP (*P*=0.0188, Mann–Whitney *U-*test). In contrast, there was no significant difference in AFP concentrations between CD8 T-cell responders and non-responders (Figure 3A–C). Median serum AFP concentrations were 7.5-fold higher in HCC patients who lacked a CD4 T-cell response (non-responders) than in those who had a CD4 T-cell response (responders) (Figure 3B). There was also a significant difference in the number of patients with Okuda tumour stage I *vs* those with stage II or III in CD4 T-cell responders (*χ*^2^, *P*=0.04; 95% confidence intervals), whereas there was no difference in the number of patients with Okuda tumour stage I *vs* stage II or III in CD8 or total T-cell responders. This indicates that patients in stage II or III are significantly less likely to have an anti-AFP Th1 response, whereas anti-AFP Tc1 responses remain unchanged.

**Figure 4:**
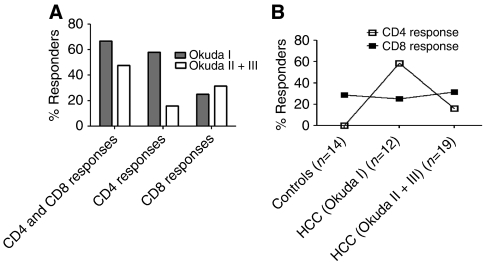
Anti-AFP CD4 T-cell response is more likely to be detected in HCC patients with early stage of disease. (**A**) The percentages of anti-AFP T-cell response (both CD4 and CD8), CD4 T-cell response or CD8 T-cell response are shown in HCC patients with different stage of disease (Okuda I *vs* Okuda II+III). (**B**) The percentages of anti-AFP CD4 or CD8 T-cell responses in controls, HCC patients with Okuda I and HCC patients with Okuda II+III are shown.

Overall, the results demonstrate that CD8 T-cell responses to AFP are detectable in control groups (28.5%), as well as in Okuda tumour stage I HCC patients (25%) and Okuda tumour stage II+III HCC patients (31.6%). In contrast, AFP-specific CD4 T-cell responses are only detected in HCC patients and response was mainly detected in Okuda tumour stage I HCC patients (58.3%) and to a lesser extent in Okuda tumour stage II or III HCC patients (15.8%) (Figure 4B).

### The balance between anti-AFP Th1 and Tc1 responses switches to an anti-AFP Tc1 response as liver cirrhosis progresses

**Figure 5:**
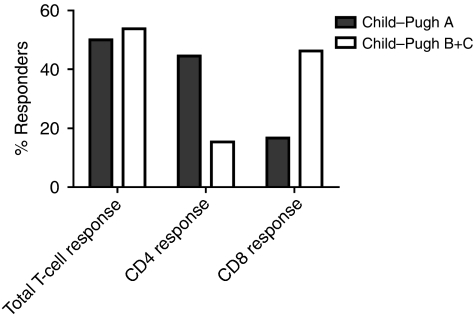
Th1 response is mainly detected in HCC patients with Child–Pugh A grading. The percentages of anti-AFP T-cell response (both CD4 and CD8), CD4 T-cell response or CD8 T-cell response are shown in HCC patients with different Child–Pugh scores (A or B+C).

Child–Pugh grading is the most commonly used method to evaluate liver cirrhosis and liver function in HCC patients, and is used to assess prognosis, evaluate the required strength of treatment required and determine the necessity for surgical intervention. Of the 31 HCC patients assessed, 18 were classified as having a Child–Pugh A score and 13 patients were classified as having a Child–Pugh B or C score. We analysed the presence of AFP-specific CD4 and CD8 T-cell responses in HCC patients with these different Child–Pugh scores. Just over 44% of HCC patients with a Child–Pugh A score had a CD4 T-cell response (8 out of 18), whereas only 15% of HCC patients with a Child–Pugh B or C score had as CD4 T-cell response (2 out of 13), suggesting that an AFP-specific Th1 response is mainly detected in HCC patients with a Child–Pugh A score (Figure 5). A CD8 T-cell response was detected in 17% of HCC patients with a Child–Pugh A score (3 out of 18), and in 46% of HCC patients with a Child–Pugh B or C score (6 out of 13, Figure 5). These results suggest that, in contrast to a Th1 response, a Tc1 response is mainly detected in HCC patients with a Child–Pugh B or C score. However, total T-cell response (CD4 and CD8) was detected in 50 (9 out of 18) and 53% (7 out of 13) of HCC patients with a Child–Pugh A score and a Child–Pugh B+C score, respectively. Taken together, these results suggest that the balance between anti-AFP Th1 and Tc1 responses switches to an anti-AFP Tc1 response as liver cirrhosis progresses.

## Discussion

There are contrasting reports on the presence of anti-AFP T-cell responses and their association with clinical characteristics of patients, such as serum AFP concentration and the stage of disease in HCC patients (Ritter *et al*, 2004; Alisa *et al*, 2005; Ayaru *et al*, 2007; Butterfield *et al*, 2007; Evdokimova *et al*, 2007; Thimme *et al*, 2008). We hypothesised that these contrasting reports may be due to a difference in the presence of anti-AFP CD4 and CD8 T-cell responses over the course of disease. A concomitant analysis of anti-AFP CD4 and CD8 T-cell responses in the same group of patients showed that anti-AFP CD4 T-cell responses are present in HCC patients but not in controls, whereas anti-AFP CD8 T-cell responses are present in both HCC patients and controls. CD4 T cells from HCC patients reacted to a wide range of peptide pools, suggesting that the anti-AFP CD4 T-cell response is multi-specific (data not shown). However, owing to the limited number of cells isolated from HCC patients, we were unable to analyse the responses to individual peptides in all patients with a T-cell response. For the same reason, we were also unable to analyse the *ex vivo* responses in these groups of patients. However, we have previously reported that AFP-specific CD4 T-cell responses are not detectable *ex vivo* (Ayaru *et al*, 2007).

In this study, we used a panel of 9–10-mer peptides that are normally expected to bind to MHC class I molecules. However, we showed that 10 mer AFP-derived peptides can also be presented by MHC class II molecules, and that CD4 T cells could respond equally well to 14- or 10-mer peptides and produce IFN-*γ* (Alisa *et al*, 2005). AFP-specific Th1 cells may produce IFN-*γ*, IL-2 and TNF-*α* (Alisa *et al*, 2005). Interleukin-2 is often associated with a memory response, and it can be produced by both antigen-specific CD4 and CD8 T cells. In our experience, not all AFP-specific IFN-*γ* producing T cells produced detectable intracellular IL-2. Thus, we decided to analyse the expansion of antigen-specific Th1 cells that are characterised by IFN-*γ* production.

It has been suggested that healthy donors have a weak response to the whole AFP protein and this response is only detectable when the antigen is presented by dendritic cells (DCs) (Evdokimova *et al*, 2007). The presentation of peptide epitopes to T cells by DCs has been shown to amplify the underlying response and improve the sensitivity of assays (Whelan *et al*, 2009). This indicates that a weak anti-AFP Th1 response is present in healthy donors and that the detection of this response would require professional antigen-presenting cells. Accordingly, we were able to detect IFN-*γ-*producing AFP-specific CD8 T cells in healthy donors. In contrast to our finding, Mizukoshi *et al* (2005) were unable to detect this response in healthy donors. This discrepancy could be explained by the methods used in different studies, with *ex vivo* assays detecting effector and expanded T cells detecting memory populations. Furthermore, a comprehensive and careful analysis of anti-AFP CD8 T-cell responses by Thimme *et al* (2008) demonstrated that anti-AFP CD8 T-cell responses are detectable in controls and in HCC patients (Thimme *et al*, 2008).

The results from several studies support the fact that the presence of AFP-specific CD8 T cells does not correlate with clinical features of disease such as elevated serum AFP concentrations (Ritter *et al*, 2004; Butterfield *et al*, 2007; Thimme *et al*, 2008). Moreover, no association has been observed between the concentration of serum AFP in HCC patients and the presence of CD8 T-cell responses to non-AFP tumour-associated antigens, such as NY-ESO-1 (Gehring *et al*, 2009). Our results support these findings and demonstrate that anti-AFP Tc1 responses are detected in all groups of patients. In contrast, anti-AFP Th1 responses are more likely to be detected in HCC patients in an early stage of disease and in cases in which the concentration of serum AFP is low. This suggests that there is a difference in the activation of anti-AFP CD4 *vs* CD8 T cells in HCC patients, with a CD4 T-cell response expanding in early stages of disease, which is usually associated with low concentrations of serum AFP, and with exhaustion of this response in later stages of disease in which there is a high concentration of serum AFP. This is in accordance with our earlier reports showing that high concentrations of AFP suppress immune cell function *in vitro* (Um *et al*, 2004), and CD4 T cells isolated from HCC patients with high concentrations of serum AFP are impaired (Alisa *et al*, 2005). Our results demonstrate that Th1 response was mainly detected in HCC patients with a Child–Pugh A score and Tc1 response was largely detected in HCC patients with a Child–Pugh B or C score. To our knowledge, this is the first report suggesting that the balance between anti-AFP Th1 and Tc1 responses switches to an anti-AFP Tc1 response as liver cirrhosis progresses. However, it is not clear why Tc1 response is preferentially expanded in patients with severe liver cirrhosis.

In conclusion, these results suggest that anti-AFP Th1 responses are more likely to be present in an early disease stage, whereas anti-AFP Tc1 responses are more likely to be present when liver cirrhosis has developed. We believe that the results presented in this paper will advance our understanding of anti-tumour immune response in HCC patients and will therefore have important implications for the development of vaccines or vaccination strategies.

## Figures and Tables

**Table 1:** Hepatocellular carcinoma (HCC) patients evaluated in this study

**Sample ID**	**Gender**	**Age (years)**	**Cirrhosis**	**Tumour**	**Hepatitis**	**Okuda^a^**	**AFP** **(ng ml^−1^**)	**Child–Pugh**
HCC01	M	62	+	Multifocal	HCV	III	180	B
HCC02	M	62	+	Single	HCV	I	8	A
HCC03	M	60	+	Single	HBV/HCV	II	200	B
HCC04	M	53	+	Single	HBV/HCV	II	37	B
HCC05	M	63	+	Multifocal	HCV	I	64	A
HCC06	M	66	+	Multifocal	HCV	I	32	A
HCC07	M	66	+	Multifocal	HCV	I	10	A
HCC08	M	55	+	Multifocal	—	II	145	C
HCC09	M	66	+	Multifocal	HBV	I	8	A
HCC10	M	52	+	Single	HBV	II	48	B
HCC11	M	55	−	Multifocal	—	I	60	A
HCC12	M	63	+	Diffuse	—	I	13 077	A
HCC13	M	35	+	Multifocal	HBV/HCV	III	96 730	B
HCC14	M	59	+	Single	—	I	41 930	A
HCC15	F	32	+	Multifocal	HBV	II	31	A
HCC16	F	58	+	Multifocal	HBV/HCV	I	14	A
HCC17	M	67	+	Multifocal	HBV	II	10	A
HCC18	M	32	+	Multifocal	—	I	8	A
HCC19	F	71	+	Multifocal	HBV	II	813 500	A
HCC20	M	79	+	Multifocal	HCV	III	70	C
HCC21	M	61	-	Diffuse	HCV	II	1234	B
HCC22	M	61	+	Multifocal	—	III	5	C
HCC23	M	62	+	Multifocal	HBV/HCV	II	1501	B
HCC24	M	67	+	Diffuse	HBV	II	188	A
HCC25	M	84	+	Single	HCV/HBV	I	1	A
HCC26	F	67	+	Diffuse	HBV	II	12 432	B
HCC27	M	62	+	Single	HBV/HCV	II	165	C
HCC28	F	46	+	Single	HBV/HCV	II	10	A
HCC29	M	54	+	Single	HCV	II	14	B
HCC30	M	53	+	Multifocal	HCV	II	157	A
HCC31	M	58	+	Multifocal	HCV	I	108	A

Abbreviations: AFP=*α*-fetoprotein; F=female; HBV=Hepatitis B virus; HCV=Hepatitis C virus; M=male.

a Okuda is clinical staging systems for hepatocellular carcinoma.

**Table 2:** Clinical characteristics of the controls

**Variables**	**All controls (*n*=14)**
*Patients with liver cirrhosis n*=*8*	
Median age (years)	56.5 (34–75)
Patients with HCV or HBV/others	4/4
Median AFP (ng ml^−1^)	9.5 (4–11)
	
*Healthy donors n*=*6*	
Median age (years)	35.8 (23–42)
AFP (ng ml^−1^)	Unknown

Abbreviations: AFP=*α*-fetoprotein; HBV=Hepatitis B virus; HCV=Hepatitis C virus.

## Supplementary Material

Supplementary Figure Legends

Supplementary Table 1S
