# Supplementary figures and images for: Expansion of anti-AFP Th1 and Tc1 responses in hepatocellular carcinoma occur in different stages of disease
Source: Br J Cancer. 2010 Jan 19;102(4):748–53. doi: 10.1038/sj.bjc.6605526 (PMC2837570; doi:10.1038/sj.bjc.6605526)

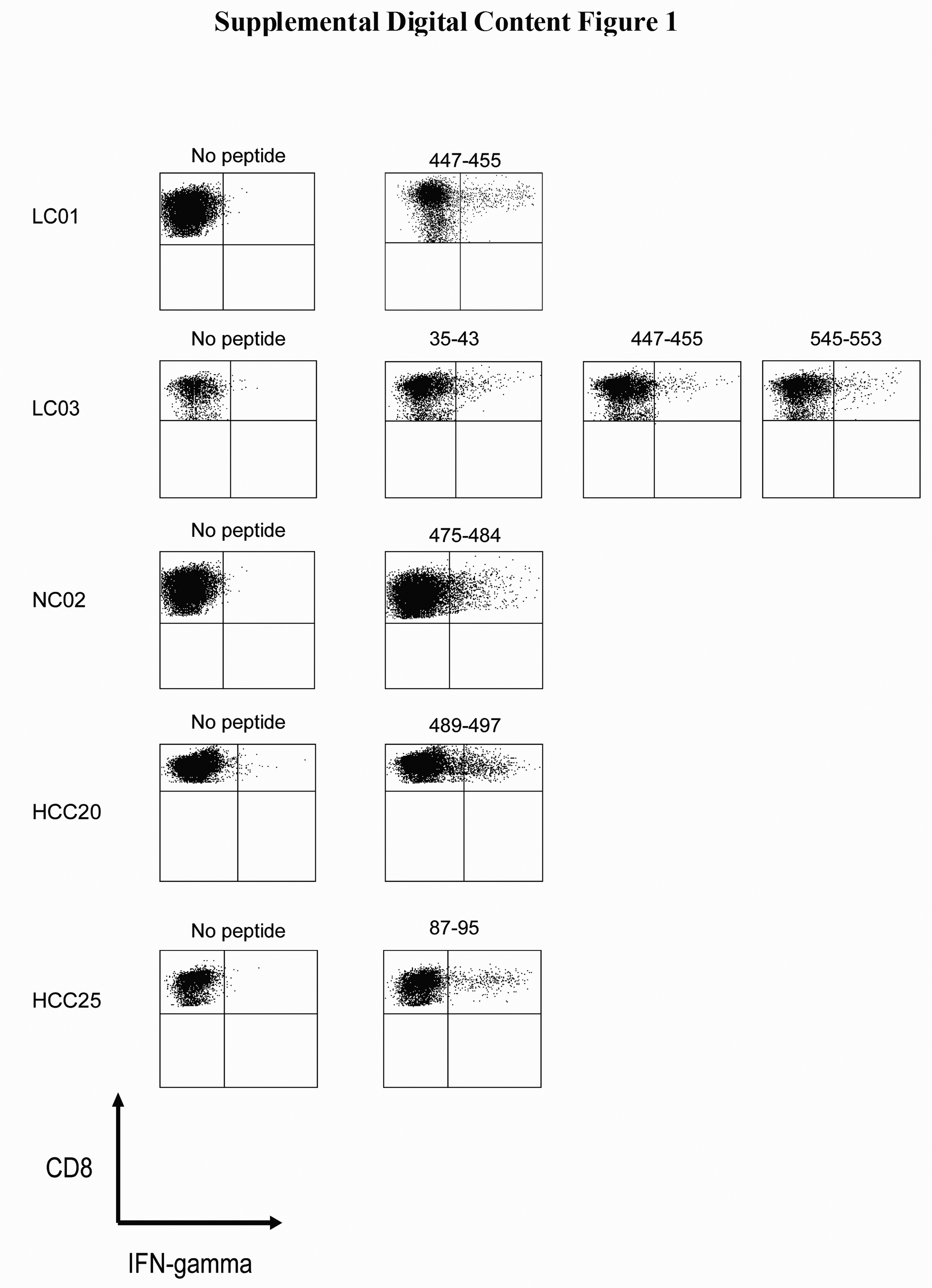

Supplement: Supplementary Figure 1 [file 6605526x1.tif]

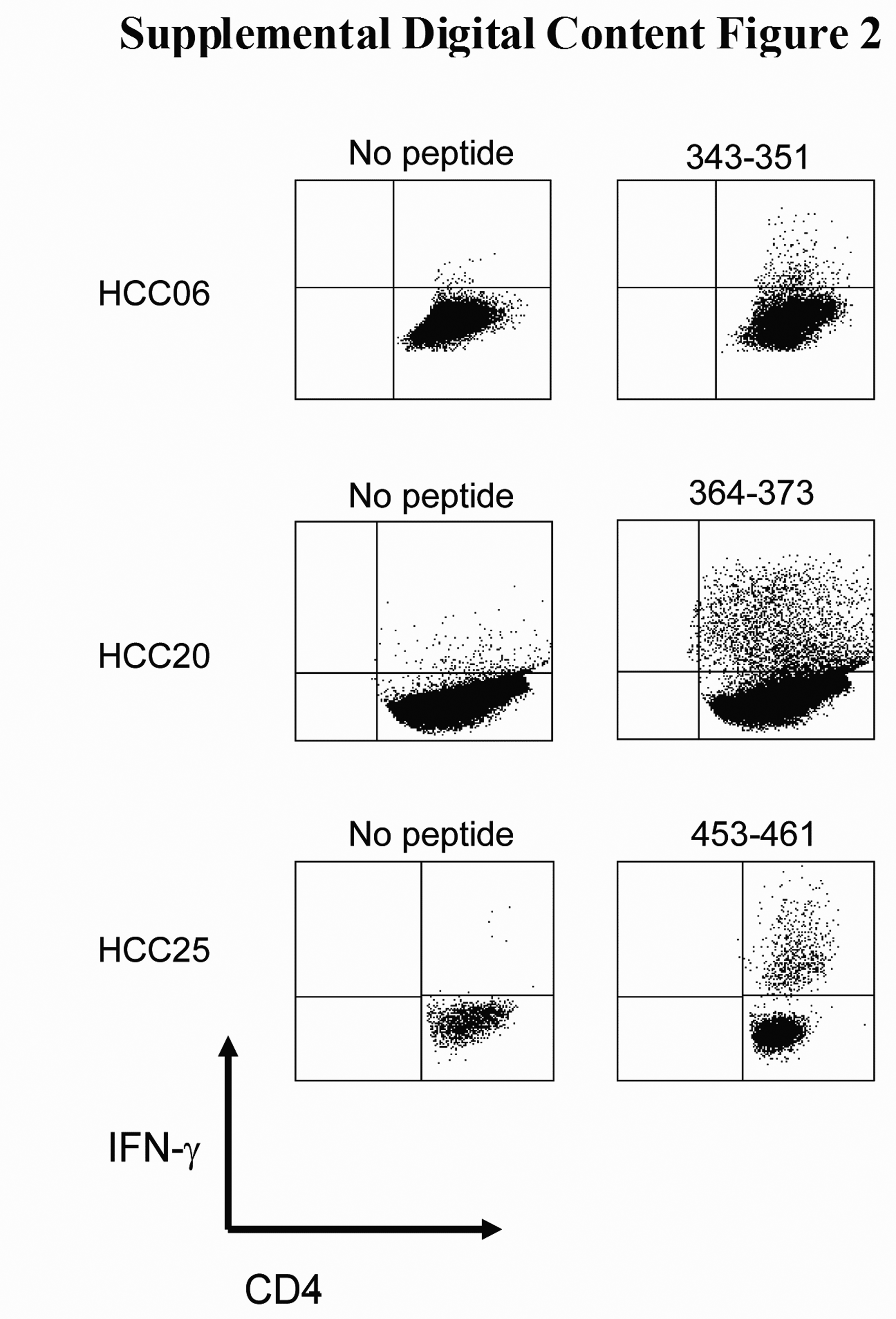

Supplement: Supplementary Figure 2 [file 6605526x2.tif]
