# Supplementary material for: Expansion of anti-AFP Th1 and Tc1 responses in hepatocellular carcinoma occur in different stages of disease
Source: Br J Cancer. 2010 Jan 19;102(4):748–53. doi: 10.1038/sj.bjc.6605526 (PMC2837570; doi:10.1038/sj.bjc.6605526)
Supplement: Supplementary Figure Legends [file 6605526x3.doc]

**Supplementary Figure 1: CD8 T cells isolated from HCC patients and controls recognize AFP-derived peptides and produce IFN-gamma.** PBMCs of patients with non-HCC liver disease (LC01 and LC03), normal control (NC02) and HCC patients (HCC20 and HCC25) were cultured in vitro to expand T cells. The expanded T cells were re-stimulated with individual peptides and the expression of IFN-gamma in CD8 T cells was analysed using an intracellular cytokine assay.

**Supplementary Figure 2: CD4 T cells isolated from HCC patients recognize AFP-derived peptides and produce IFN-gamma.** PBMCs of HCC patients (HCC06, HCC20 and HCC25) were expanded in vitro in the presence of rIL-2 and AFP-derived peptides. The expanded T cells were re-stimulated with individual peptides and the expression of IFN-gamma in CD4 T cells was analysed using an intracellular cytokine assay.
