# Supplementary material for: Expansion of anti-AFP Th1 and Tc1 responses in hepatocellular carcinoma occur in different stages of disease
Source: Br J Cancer. 2010 Jan 19;102(4):748–53. doi: 10.1038/sj.bjc.6605526 (PMC2837570; doi:10.1038/sj.bjc.6605526)
Supplement: Supplementary Table 1S [file 6605526x4.doc]

Supplemental Digital Content

| **Amino Acid Start** | **Sequence** | **Amino Acid Start** | **Sequence** |
| --- | --- | --- | --- |
| 1 | MKWVESIFL | 347 | KNIFLASFV |
| 4 | VESIFLIFL | 350 | FLASFVHEY |
| 8 | FLIFLLNFT | 364 | QLAVSVILRV |
| 11 | FLLNFTESRT | 372 | RVAKGYQEL |
| 20 | TLHRNEYGI | 379 | ELLEKCFQT |
| 30 | SILDSYQCTA | 385 | FQTENPLEC |
| 35 | YQCTAEISL | 410 | ALAKRSCGL |
| 37 | CTAEISLADL | 419 | FQKLGEYYL |
| 40 | EISLADLATI | 427 | LQNAFLVAYT |
| 46 | LATIFFAQFV | 431 | FLVAYTKKA |
| 47 | ATIFFAQFV | 441 | QLTSSELMAI |
| 54 | FVQEATYKEV | 447 | LMAITRKMA |
| 65 | KMVKDALTAI | 449 | AITRKMAAT |
| 70 | ALTAIEKPT | 453 | KMAATAATCC |
| 86 | CLENQLPAFL | 462 | CQLSEDKLL |
| 87 | LENQLPAFL | 468 | KLLACGEGA |
| 89 | NQLPAFLEEL | 475 | GAADIIIGHL |
| 125 | FLAHKKPTPA | 485 | CIRHEMTPV |
| 137 | PLFQVPEPV | 489 | EMTPVNPGV |
| 140 | QVPEPVTSC | 492 | PVNPGVGQC |
| 158 | FMNKFIYEI | 498 | GQCCTSSYA |
| 164 | YEIARRHPFL | 507 | NRRPCFSSLV |
| 172 | FLYAPTILL | 514 | SLVVDETYV |
| 174 | YAPTILLWA | 531 | FIFHKDLCQA |
| 179 | LLWAARYDKI | 536 | DLCQAQGVAL |
| 187 | KIIPSCCKA | 542 | GVALQTMKQ |
| 217 | SLLNQHACAV | 545 | LQTMKQEFLI |
| 235 | FQAITVTKL | 546 | QTMKQEFLI |
| 249 | KVNFTEIQKL | 548 | MKQEFLINL |
| 277 | CLQDGEKIM | 555 | NLVKQKPQI |
| 298 | KITECCKLTT | 562 | QITEEQLEAV |
| 306 | TTLERGQCII | 570 | AVIADFSGL |
| 325 | GLSPNLNRFL | 576 | SGLLEKCCQ |
| 343 | SSGEKNIFL | 598 | KLISKTRAAL |

Table 1S. Alpha-fetoprotein derived peptides.
